# Improving Recycled Aggregate Quality by Mechanical Pre-Processing

**DOI:** 10.3390/ma13194342

**Published:** 2020-09-29

**Authors:** Madumita Sadagopan, Katarina Malaga, Agnes Nagy

**Affiliations:** 1 Department of Resource Recovery and Building Technology; University of Borås, 50190 Borås, Sweden; katarina.malaga@ri.se (K.M.); agnes.nagy@hb.se (A.N.); 2 RISE, CBI Swedish Cement and Concrete Research Institute, 50115 Borås, Sweden

**Keywords:** concrete recycling, sustainability, recycled aggregates, mechanical pre-processing, aggregate quality, packing density, aggregate shape, compressive strength, workability

## Abstract

Concrete with crushed concrete aggregates (CCA) shows lesser compressive strength than reference concrete with natural aggregates. The goal of this study is to improve the strength of structural concrete with 53% and 100% CCA replacements without increasing the cement content. Thus, improvements in CCA quality are induced by combining mechanical and pre-soaking pre-processing techniques. Mechanical pre-processing by rotating drum is separately pursued on fine and coarse CCA for 10 and 15 min respectively. Results show, adhered mortar content and CCA water absorption reduces as pre-processing duration increases. Pre-processing influences CCA particle grading, flakiness index, shape index, void-content, unit-weight and density, jointly seen as packing density, which increases with pre-processing duration. Water amount to pre-soak CCA before concrete mixing is stable despite grading modifications, due to reduced water absorption resulting from mechanical pre-processing. Compressive strength and workability for pre-processed CCA50 and CCA100 concrete are comparable to reference concrete and show similar trends of improvement with packing density. Packing density markedly shows the quality improvements induced by pre-processing on CCA, maybe considered as one of the quality assessment indexes for CCA. Packing density should be investigated for other recipes to see the stability of the trend with workability and compressive strength.

## 1. Introduction

Crushed concrete aggregates (CCA) originating from structural concrete completes a closed-loop recycling when reused again in structural concrete. Using CCA originating from structural applications for low-utility applications, such as road backfilling, degrades the material value of CCA. By quality improvements, CCA is improved to suit high-utility applications like structural concrete, thus retaining material value. The efforts for the CCA quality improvements are necessary to channel larger amounts of CCA for structural concrete production, which as of 2018 is only 6% of the total 2.38 million tons of mineral waste from the construction sector in Sweden [1].

Several studies show that by improving CCA quality the mechanical properties of the CCA concrete can match up to the properties of concrete with gravel or crushed rock—which is usually the parent concrete for the CCA. The quality improvements in coarse CCA fractions by mechanical pre-processing are shown to produce concrete with similar compressive strength as concrete containing aggregates of crushed stone or gravel [2].

Quality of CCA is affected by the surface-adhered mortar giving CCA a higher porosity than natural gravel and crushed rock [3]. A high adhered mortar content leads to CCA with low density and high water absorption which negatively influences the concrete strength [4]. There are different methods presented in literature for improvement of CCA quality by the removal of adhered mortar such as mechanical [5,6], thermo-mechanical [5], and chemical methods by acid dissolution [7]. These methods improve the quality of CCA towards higher density and lower water absorption [5,6] leading to an improvement in concrete mechanical properties. Compressive strength of CCA concrete is shown to improve by 9% on mechanical pre-processing of coarse CCA by autogenous cleaning [8]. Another study shows the compressive strength of CCA concrete to increase by 6% and 15% for low-and medium-strength concrete recipes when the coarse CCA is pre-processed by an additional crushing stage [9].

The mechanical preprocessing techniques cause abrasion of CCA by milling either in a stationary drum containing a paddle on a rotating axis or in a rotating drum. Equipment such as eccentric rotor mill [2] has a rotating cylinder whereas the screw abrading crusher [10] has a rotating shaft-paddle. Alternatively, a rotating drum fitted with lifting plates is used for pre-processing CCA, referred to in literature as an autogenous cleaning device [8] or dry milling [11]. The CCA is treated in a horizontal rotating drum (container) lined with lifting plates causing CCA abrasion by free fall of the material.

The mechanical preprocessing of concrete rubble results in two major effects: firstly, abrasion of adhered mortar and secondly the comminution of low-density CCA or the abrasion of aggregate material from high density CCA. Screw abrading crusher and eccentric mill are seen to cause comminution of about 70% of the low-density CCA, reducing it to fine fractions [5]. With autogenous cleaning, just 10% of the coarse CCA fractions is comminuted on mechanical pre-processing [5]. Additionally, the latter shows more than 50% reduction in the CCA water absorption without significant changes to aggregate size and is therefore in comparison to other methods more effective in adhered mortar removal. Two parameters are of importance with mechanical pre-processing methods like autogenous cleaning: the pre-processing duration and the drum rotation speed, which varies from equipment to equipment. Studies show similar results on the effects of pre-processing for rotation speed within a range of 50–69 rpm and a pre-processing duration between 10 and 15 min [8,12]. Therefore, the speed and duration range is considered optimum for CCA quality improvement.

Mechanical pre-processing in literature are either carried out on coarse [8,10] or fine CCA [11] and depending on the extent of CCA substitution both coarse and fine fractions. Studies showing full replacement of coarse and fine aggregates with CCA are not pursuing mechanical pre-processing on these fractions [13].

The mechanical pre-processing of CCA is followed by washing on sieve to remove the abraded mortar particles to densify the CCA [14]. Mechanical pre-processing with autogenous cleaning includes washing of coarse CCA [8]; however, there are methods which do not wash CCA after pre-processing. The fine CCA pre-processed by dry milling in a mortar study are not washed because the pre-processing is pursued to bring the CCA particle grading closer to the reference mortar grading [11], which may influence water absorption as the detached adhered mortar is not removed by washing. Another study adopts washing procedure on fine CCA—the solely replaced fraction in the concrete, giving increased density and rounder shape therefore improved aggregate quality [15].

Mechanical pre-processing by primary crushing causes the particle grading of fine CCA to be distant from natural aggregates [16,17]. Mechanical pre-processing is induced to result in CCA particle grading closer to the reference aggregate grading [8], specifically for fine CCA by dry milling [11]. The particle grading of the coarse CCA is not distinctly improved by mechanical pre-processing after crushing as it is already of good fit. However, aggregate density is improved by adhered mortar removal in the case of high density CCA, 2640 kg/m^3^ [17], in which case mechanical pre-processing causes more abrasion of the adhered mortar than material. For lower density coarse CCA, 1946 kg/m^3^, mechanical pre-processing after crushing results in an improved particle grading towards reference aggregate grading [8] as not just the adhered mortar is removed but material abrasion also occurs and increases the amount of fine material in the aggregate fraction. Since matching grading curves yields concrete of similar workability [18], the closing in of CCA grading curves to the reference aggregate by mechanical pre-processing is seen as a method to improve CCA quality.

Mechanical pre-processing additionally influences the CCA shape as seen in studies that use multiple crushing stages to pre-process the CCA to acquire a rounder shape similar to reference aggregates [19,20]. The shape improvements are characterized by flakiness index and unit weight of aggregates [21,22,23]. Flakiness and shape index properties measure the width–length proportions and the thickness–length proportions, respectively [24]. The flakiness index investigates the shape of a sample of aggregates by sieving whereas the shape index is tested on individual aggregate particles by a special caliper.

Mechanical pre-processing influences properties—such as density, flakiness index, shape index, and particle grading—prominent in assessing aggregate quality for structural concrete according to standards SS-EN 12620 [25]. These properties are of technical importance because they jointly contribute to the dense packing of coarse and fine aggregates in the concrete, known as packing density [8,26]. Previous research shows that enhanced packing density contributes to good workability and improved compressive strength [8,27]. Packing density is a weighed sum of the individual packing densities of fine and coarse fractions tested separately as unit weight [28]. Like packing density, the unit weight is influenced by the shape, grading, and density of aggregate and measures the bulk mass of aggregates including the voids in between the aggregate particles in a fixed volume [24]. The unit weight test also measures the void content formed between the aggregate particles for the investigated fraction.

Improvements in flakiness index and particle grading induced by crushing methods are shown to increase the concrete compressive strength and workability [8,22], also contributing to packing density. Alternatively, packing density is improved by modifying aggregate grading based on the unit weights of aggregate fraction with the aim of increasing concrete compressive strength [28,29].

Besides improving CCA density by mechanical pre-processing, there exist other pre-processing methods in literature that address the water absorption need of CCA, pre-processing by pre-soaking CCA with water [17,30]. A combination of these two pre-processing methods further improves the quality of the CCA concrete [8].

The novelty of this study is the mechanical pre-processing of both fine and coarse CCA to induce adhered mortar removal in comparison to previous literature where pre-processing is carried out in either coarse or fine fractions.

A drum fitted with ribs with a rotating speed of 50 rpm is used to mechanically pre-process fine and coarse CCA separately in laboratory conditions. The mechanical pre-processing is evaluated for two durations of 10 and 15 min, considered optimum by literature for the CCA investigated in the respective studies [8,11]. Moreover, previous research conducts pre-processing on either coarse or fine aggregates for a specified duration [8,11]. This study chooses two durations assuming that different effects on coarse or fine fraction might arise from the same pre-processing duration; moreover, this approach gives the best suiting pre-processing duration specific for fine and coarse CCA fractions.

The pre-processed aggregates are washed on sieves to remove the abraded adhered mortar or aggregate material. The pre-processing efficiency is determined firstly by the amount of adhered mortar loss along with the material damage occurring on CCA. This can be seen as a new contribution to literature, which solely addresses the adhered mortar loss in CCA by pre-processing. The improvements in CCA properties—such as density, water absorption, flakiness index, shape index, unit weight, void content, and packing density—are also investigated on pre-processed CCA.

Concrete with mechanically pre-processed CCA is investigated for 100% (CCA100) and 53% (CCA50) replacements in the reference concrete recipe. The CCA replacement percentages aim to address practical issues such as the availability of waste and environmental issues such as exploitation of natural resources. CCA100 fits for a continuous supply of concrete waste, substitutes coarse and fine natural aggregates, thus preventing further waste. The replacement of fine aggregate fraction with CCA, as in CCA50, substitutes natural sand, which is critical for ecological preservation and a concern for Swedish concrete producers, who have already transitioned towards substituting coarse natural aggregates with crushed stone. From a logistics perspective, CCA50 alternative fits scarce concrete waste supply to make possible replacements on a continuous basis.

The mechanical preprocessing of CCA is combined with pre-processing of CCA by presoaking based on the water absorption of the CCA. The mechanical pre-processing improves the density of the CCA by removal of adhered mortar resulting in a lower water absorption, which consequently reduces the amount of pre-soaking water. The CCA properties jointly influence the aggregate packing density, which in turn influences the compressive strength and workability of the concrete. Packing density concludes the collaborative effect of several aggregate properties.

## 2. Materials and Methods

**Figure 1:**
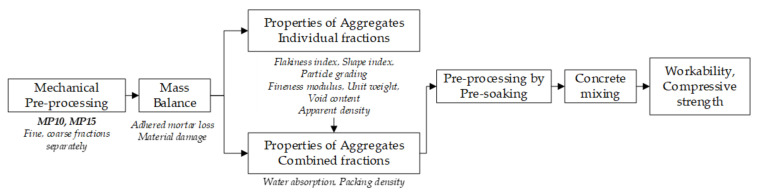
Experimental scheme.

The experimental program, see Figure 1, is commenced with mechanical pre-processing alternative for individual fractions followed by an assessment of adhered mortar loss and material damage on a single batch sample. The properties of individual and combined fractions are investigated on pre-processed CCA. Investigated properties and samples per test covered in Section 2.2.

The CCA investigated in this study originates from the prefabricated rejects of an industrially active concrete recipe of grade C40/50, also the reference concrete recipe in this study. In the reference concrete recipe, the CCA replaces:

1. All fine aggregates corresponding to 53% of total aggregate content (CCA50)
2. 100% of coarse and fine aggregates (CCA100)

The reference aggregates are crushed stone of size 8/11.2 mm and naturally graded aggregate 0/8 mm. The CCA fractions are 8/11.2, 0.5/4, and 0/4 mm; fine CCA fractions are differently sized from reference aggregates as after crushing the particle grading has been modified to fit reference concrete grading [17]. The 8/11.2 mm fraction is produced by crushing concrete rubble in a jaw crusher with openings of 12 mm and sieved. Fine CCA fractions 0/4 mm and 0.5/4 mm are crushed at 4 mm opening and sieved. In order to compare and control the losses from mechanical pre-processing, the 0/8 mm natural aggregate is split into fractions 0.5/4 and 0/4 mm.

### 2.1. Mechanical Pre-Processing of Aggregates

The mechanical pre-processing is based on autogenous cleaning method carried out in a metal drum rotating at 50 rpm, tilted horizontally at an angle of 18° and is lined with vertical ribs to facilitate material lift and cause abrasion by free fall. The coarse and fine aggregates are individually pre-processed and the aggregate quantity in a single batch corresponds to a third of the drum volume, the drum having a height of 30 cm and diameter 27 cm. The aggregate quantity is weighed before pre-processing to determine the loss by pre-processing.

Two mechanical pre-processing alternatives with durations of 10 and 15 min denoted as MP10 and MP15 respectively are investigated. In addition, un-preprocessed aggregates, denoted by MP0, are also investigated. After pre-processing the aggregates are washed on sieves to remove the displaced material or adhered mortar. The sieve mesh size used for washing depends on aggregate fraction size, shown in Table 1. The aggregates are subsequently dried to room temperature and weighed. The reference aggregates are also pre-processed in order to identify the effect of mechanical pre-processing on material loss caused by abrasion. In order to make an effective comparison of pre-processing losses of CCA and reference aggregates, the 0/8 natural aggregate is split into fractions 0.5/4 and 0/4 mm for pre-processing.

The loss of adhered mortar caused by mechanical pre-processing is assessed from the change in adhered mortar content before and after pre-processing. The adhered mortar content on an aggregate fraction is experimentally determined by a thermal shock resulting in the displacement of adhered mortar from the aggregate surface [8,31]. The test is pursued on two samples of 100 g each where aggregate sample of weight (m_initial_) is soaked in water for a duration of 2 h and subsequently heated at a temperature of 500 °C for 2 h followed by immediate immersion in 20 °C water. The aggregates are finally oven-dried and their weight is measured as m_final_. The adhered mortar loss is calculated according to Equation (1).

(1) $$\mathrm{Adhered}\;\mathrm{mortar}\;\mathrm{loss}\;\mathrm{by}\;\mathrm{thermal}\;\mathrm{shock}\;=\;\frac{m_{\mathrm{initial}}-m_{\mathrm{final}}}{m_{\mathrm{initial}}}\;\left(\%\right)$$

The losses from the mechanical pre-processing alternatives MP10 and MP15 are analyzed for reference and CCA concrete aggregates. After mechanical pre-processing, the water absorption of the combined CCA fraction is tested by the modified pycnometer method [17] to determine the amount of water added to pre-process the CCA by pre-soaking just before mixing.

### 2.2. Properties of Aggregates

The CCA and reference aggregates are characterized by properties shown in Table 2 before and after the mechanical pre-processing alternatives MP10, MP15 to analyze changes in aggregate quality with pre-processing; MP0 denotes un-preprocessed alternative.

The flakiness index of fine aggregates is investigated down to 1 mm by a Swedish method developed to characterize crushed rock aggregates [33].

The packing density is derived for all concrete mixes—reference concrete, CCA50 and CCA100 in this study.

### 2.3. Casting Concrete with Mechanically Preprocessed Aggregates

The cement used in this study is CEM II/A-LL 42.5 R, and the superplasticizer is polycarboxylate-based with a dry content of 24 ± 1 weight %. The reference concrete has a water–cement ratio of 0.5 and is constant for CCA100 and CCA50 concrete mixes.

Before mixing the CCA are pre-processed by momentarily pre-soaking with water based on the 15 min water absorption value of the combined CCA fraction determined by the modified pycnometer method [17]. Since the pre-soaking is meant to saturate the aggregates, it is not accounted for in the water–cement ratio. In the CCA concrete mixing process, the mixing water is added in two-stages so that the CCA–cement paste interface commences to strengthen while mixing is ongoing [17]. Concrete recipes for the different mixes are presented in Table 3.

Following the mixing process, the workability of the fresh concrete is determined by measuring the slump and flow diameter, once per batch, according to SS-EN 12350-5. Three cylinder specimens of size 100 mm × 200 mm are cast, demolded after 24 h and cured for 28 days submerged in a curing tank with water at 20 ± 2 °C according to SS-EN 12390-2. Compressive strength is tested according to SS EN 12390-3 at 28 days.

## 3. Results and Analysis

### 3.1. Adhered Mortar Loss after Pre-Processing

Assessment of adhered mortar loss is a way to measure the effectiveness of the pre-processing alternatives of 10 and 15 min. Mechanical pre-processing is pursued separately for each CCA and reference aggregate fraction and is followed up by related operations washing and sieving. CCA may undergo both loss of material and adhered mortar, whereas reference aggregates undergo a loss of material. Therefore, the actual amount of adhered mortar removed from CCA by mechanical pre-processing is the material loss in reference aggregates deducted from the CCA loss as shown in Equation (2).

(2) $${\mathrm{adhered}\;\mathrm{mortar}\;\mathrm{loss}}_{\mathrm{fraction}}\;=\;\frac{{\mathrm{CCA}}_{\mathrm{loss}\%}-{\mathrm{reference}\;\mathrm{concrete}\;\mathrm{aggregate}}_{\mathrm{loss}\%}}{{\mathrm{reference}\;\mathrm{concrete}\;\mathrm{aggregate}}_{\mathrm{loss}\%}}\;\left(\%\right)$$

Mass balance is made to control the losses occurring in the washing and sieving. Especially when both coarse and fines are mechanically pre-processed it is of importance to ascertain that adhered mortar loss is coming up just marginally from the processes of washing and sieving but more from actual mechanical pre-processing. Mass balance is made individually for each CCA and reference aggregate fraction in three stages:

1. Thermal shock before pre-processing on each fraction to assess the potential loss of material and adhered mortar.
2. Losses by mechanical pre-processing to assess the actual material and/or adhered mortar loss
3. Thermal shock after pre-processing to determine the remaining material and adhered mortar left on the aggregate. The sum of the loss from mechanical pre-processing and thermal shock from stage 3 should only exceed by small margins the losses from thermal shock before pre-processing. The small margins validate mechanical pre-processing and related processes to give reliable results on the adhered mortar loss.

**Figure 2:**
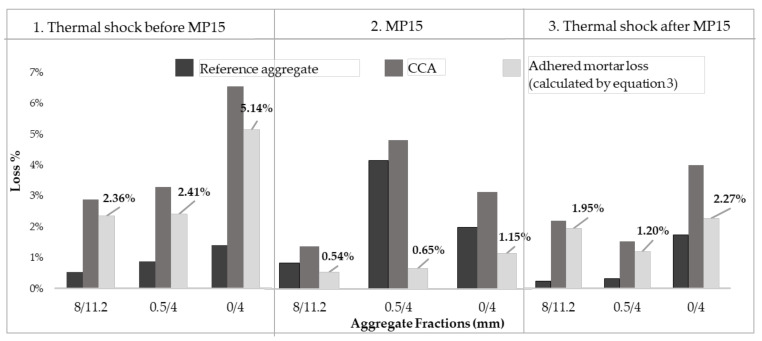
Adhered mortar loss by mechanical pre-processing MP15.

Mass balance for a coarse aggregate fraction 8/11.2 mm show that the potential adhered mortar loss given by thermal shock before pre-processing is 2.36%, Figure 2. With mechanical pre-processing MP15 including washing and sieving operations, a loss of 0.54% is observed. The final thermal shock shows 1.95% loss of mortar, which is the remaining adhered mortar on the CCA after pre-processing. The total mass balance for fraction 8/11.2 occurring in these three stages has an error of 0.13%, arising mostly from washing and sieving. For the other fractions and MP10 alternative, similar mass balances are made with an error range in between 0.02–1.9%, considered acceptable for further pursuing of testing of aggregate properties.

In stage 2 mechanical pre-processing MP15, Figure 2, the fines show higher values of adhered mortar loss compared to coarse and is consistent with literature findings [4]. The 0/4 mm fraction shows 5.14% possible adhered mortar loss at stage 1, with a loss from pre-processing of 1.15% it remains most abundant in mortar of all fractions in stage 3 with 2.27%. This will have implications on the water absorption especially for CCA50, with only fines replaced, where the reduction on water absorption after pre-processing is not so marked.

The effectiveness of alternative MP15 is assessed to at least 22% in all fractions by relating losses in mechanical pre-processing in stage 2 to losses in thermal shock at stage 1. The adhered mortar loss for 0/4 mm fraction is 22%, 0.5/4 mm is 26%, and 8/11.2 mm is 22% which makes MP15 the alternative with most uniform eroding effect for each fraction compared to MP10 where removed mortar amount shows higher variability.

### 3.2. Tested Properties of Mechanically Pre-Processed Aggregates

#### 3.2.1. Particle Grading

**Figure 3:**
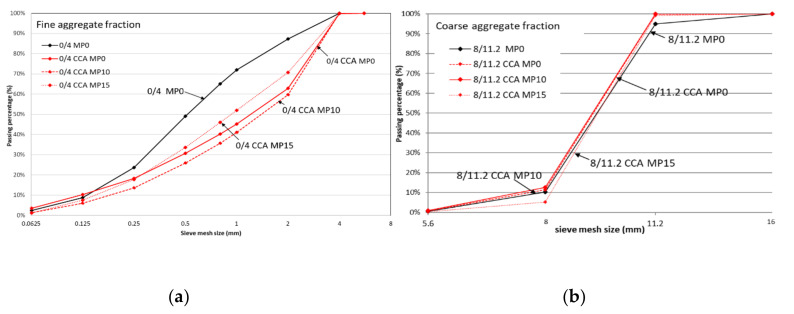
Grading curves with mechanical pre-processing (**a**) fine fraction (**b**) coarse fraction.

Grading curves are aggregate quality indicators, such that when CCA grading matches with reference concrete aggregates results in good concrete workability and compressive strength [18]. The mechanical pre-processing alternative MP15 shows a positive influence on the grading curve of the fine CCA fraction by moving closer to the reference aggregate grading, see Figure 3a. This observation is consistent with literature where grading curves of fine CCA resulting from dry milling are shown to lie close to the reference concrete grading [11]. The particle grading improvement in fines with mechanical pre-processing MP15 confirms CCA quality improvement as it improves workability at CCA50, where solely fines undergo pre-processing. The workability is in the same quality class as reference concrete workability, F2, see Figure 5.

The coarse CCA grading curve, Figure 3b, is not markedly influenced by MP10 or MP15, maybe because its higher density resists significant abrasion by mechanical pre-processing. Another study shows noticeable effect on the course fraction grading with pre-processing like autogenous cleaning as it is a low-density CCA [8].

**Figure 4:**
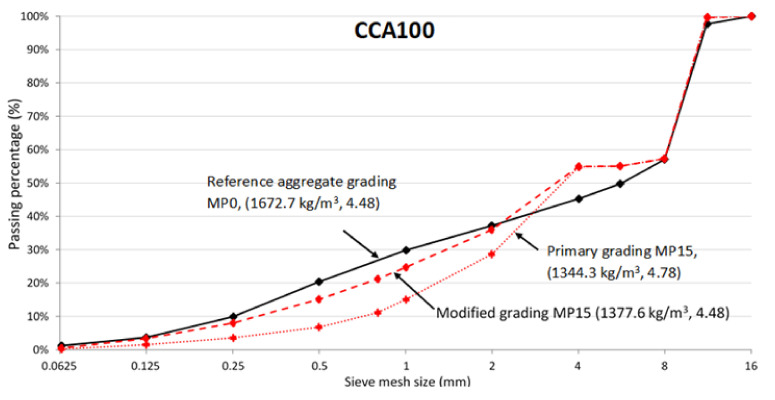
Combined grading curves CCA100 (packing density, fineness modulus).

To assess the closeness of grading curves across fine and coarse CCA fractions, combined grading curves are prepared for each CCA concrete as shown in Figure 4.

The CCA grading curve investigated directly after crushing is improved by mechanical pre-processing to come closer to the reference concrete grading curve. However, there is still some gap between the grading curve directly after pre-processing (MP15 primary grading) and the reference concrete curve. Therefore, the grading curve is modified to get closer to the reference concrete grading by re-adjusting the share of CCA fractions in the concrete recipe, CCA proportions seen in Table 4.

The closeness of the modified CCA grading and reference aggregate grading is validated by the fineness modulus, seen in Figure 4 and Table 5. After pre-processing MP15, the fineness modulus of CCA with primary grading is 4.78, already close to reference aggregate grading 4.48. However, the fineness modulus of CCA after modified grading is 4.48, an excellent match to reference aggregate grading.

#### 3.2.2. Packing Density

The packing density is derived from the weighed unit weight of the aggregate fractions in the concrete mix. The packing density is also influenced collectively by aggregate density, flakiness index, shape index, particle grading, unit weight, and void content. Therefore, the changes arising in these properties from mechanical pre-processing affects the packing density. The property majorly affecting packing density is the change in the unit weight and particle grading of mechanically pre-processed aggregates, seen in Table 4. The highest packing density is observed for MP15 for both CCA50 and CCA100.

**Figure 5:**
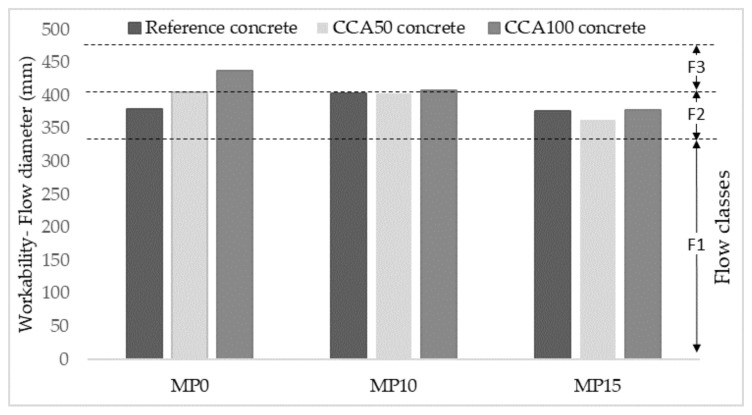
Workability of concrete mixes and standardized flow classes.

**Figure 6:**
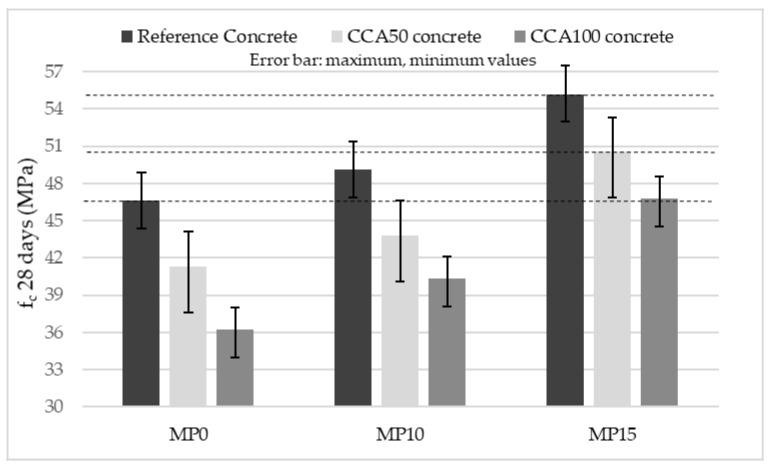
Mean compressive strength of concrete with mechanically pre-processed aggregates.

Improvements in packing density for CCA100 arises for all pre-processing alternatives and particle grading within (primary, modified) but for CCA50 arises only at MP15. Increased packing density for CCA50, with solely fines pre-processed, gives a particle grading closer to reference aggregates and leading to a better fit to reference concrete workability, Figure 5. The packing density of CCA100, where even the coarse aggregates are pre-processed, is increasing because of the increased unit weight leading to a better fit to reference concrete compressive strength, Figure 6.

In the case of reference aggregates, the packing density is seen to progressively reduce with pre-processing duration due to the material loss in the 0/8 mm fractions arising from the washing and sieving process following mechanical pre-processing.

#### 3.2.3. Water Absorption of Combined CCA Fraction

The CCA water absorption is expected to reduce with the removal of adhered mortar by mechanical pre-processing techniques, the water absorption results in this study are in agreement with this rule. The water absorption is seen to decrease after mechanical pre-processing for both CCA50 and CCA100 independent of pre-processing duration for primary graded CCA, see Table 5. The modified grading after pre-processing introduces more fines to close the gap between CCA and reference concrete grading curve causing the water absorption to increase. This increase is highest at MP10 for CCA50 and CCA100 but decrease by more than 1% is observed for CCA100 when MP15 is pursued. Therefore, for a longer pre-processing duration, the water absorption for the primary grading reduces markedly, this shows the effectiveness of the mechanical pre-processing alternative. The water absorption of modified grading with MP15 is not reduced; however, it is still lesser than the water absorption before pre-processing, especially in the case of CCA100. Mechanical pre-processing is most impactful in the reduction of water absorption when the coarse and fine aggregates are CCA such as CCA100. Fines still contain the most adhered mortar after pre-processing which is why the reduction in water absorption in primary grading is not so marked.

As the water absorption value remains unchanged for CCA100 at 5.7% with 10 min of pre-processing, the pre-soaking water amount is maintained constant in the MP0 and MP10 concrete mixes, see Table 3. Similarly, the water absorption value for CCA50 remains unchanged at 3.6% for MP10 and MP15 pre-processing alternatives thus the pre-soaking water is maintained constant for these two concrete mixes.

#### 3.2.4. Flakiness Index, Shape Index

Similar to earlier findings, the flakiness index and shape index reduce with mechanical pre-processing. Flakiness index is tested on sample, shape index on individual particles and the correlation in between them being that aggregates having a high flakiness index also have a high shape index [34]. This is the case for the coarse aggregate fraction in this study where the shape index changes correspondingly with the flakiness index for both mechanical pre-processing alternatives. For coarse CCA 8/11.2 mm, the flakiness index reduces markedly by 20% with MP10 and 40% with MP15, see Table 6. Accordingly, the shape index reduces by 15% and 20% at MP10 and MP15 respectively. Out of the two fine CCA fractions, the flakiness of 0/4 mm is clearly reducing for both pre-processing durations. The best flakiness index 6.25% for 8/11.2 CCA for MP15 occurs along with the loss of adhered mortar of 22% from pre-processing (Figure 2).

#### 3.2.5. Unit Weight and Void Content

This study investigates unit weight and void content tests in an un-compacted state in order to address the influence of shape—flakiness, shape index on unit weight [21,35]. The unit weight and void content are investigated from the same test, Equation (3).

(3) $$\mathrm{Void}\;\mathrm{content}\;=\;1-\frac{\mathrm{unit}\;\mathrm{weight}}{\mathrm{particle}\;\mathrm{density}}\times 100$$

With improved flakiness index of 8/11.2 CCA it is seen that unit weight increases gradually with each pre-processing duration. With increasing unit weight a decreasing void content is expected and this is seen for 8/11.2 CCA independent of pre-processing duration. Similarly, there is a visible reduction in the void content of fine CCA fraction 0.5/4 mm with MP10 and MP15. It is seen that this increase in unit weight for both coarse and fines is leading to an increase in the packing density and thus an improved compressive strength.

#### 3.2.6. Apparent Density

Previous research indicates that the CCA density increases with the removal of adhered mortar by mechanical pre-processing, distinctly for lower density aggregates [8]. For higher density aggregates of a normal structural concrete such as in this study, the apparent density of coarse CCA is not consistently increasing and shows relatively small changes. This is due to the loss of aggregate material happening in addition to the loss of adhered mortar, Table 6. The coarse CCA still improves concrete quality by an increased compressive strength by virtue of unit weight, Figure 6.

#### 3.2.7. Density of Hardened Concrete

The influence of the packing density on the compressive strength of concrete is initiated by a comparison of packing density with the concrete density, measured at 28 days just before the compression strength is carried out, shown in Table 6. The concrete densities of CCA50 and CCA100 increase correspondingly with the packing density as the pre-processing duration is prolonged. The concrete density is the highest at MP15 as is the packing density; however, a similar trend is not observed for reference concrete where concrete density increases despite reducing packing density. This is because the packing density in this study addresses aggregates in an un-compacted condition, whereas the apparent density and concrete density on the other hand are measured in a compacted condition. The concrete density may increase without an increase in the packing density also because of the reduced flakiness of coarse reference concrete aggregate 8/11.2 mm contributing to a more effective compaction of the fresh concrete [24].

### 3.3. Concrete Properties

#### 3.3.1. Concrete Workability

The workability of the concrete produced with mechanically pre-processed CCA for 10 and 15 min durations are measured by the flow diameter, seen in Figure 5. Both CCA50 and CCA100 is in the same quality class F2 (Flow class, SS EN 206) as reference concrete independent of pre-processing duration. The workability of CCA100 and CCA50 is improving with each pre-processing duration due to grading curve modifications having more influence on fine aggregates than on coarse aggregates to achieve a closer fitting of CCA and reference aggregate grading. Thus, a proper modification of the grading curve after mechanical pre-processing of different duration is leading to comparable workability with reference concrete. Shape improvements in coarse CCA as a result of mechanical pre-processing shown by reduced flakiness index may also positively influence workability.

#### 3.3.2. Concrete Compressive Strength

Previous research relates the increase in concrete compressive strength for mechanically pre-processed CCA to the improvements in CCA density occurring with adhered mortar removal. In this study, the CCA density is not showing a uniform response across the mechanical pre-processing alternatives despite the adhered mortar loss. However, the concrete density is uniformly increasing with pre-processing duration for both CCA50 and CCA100, Table 6. There is a definite increase in the unit weight of coarse fractions with pre-processing, reaching the highest value at MP15. The weighed unit weights of aggregate fractions stand for the packing density and thus the packing density increases with pre-processing duration. A similar trend is noticeable in mean compressive strength of CCA100, which increases with increasing pre-processing duration, see Figure 6. The increase in the compressive strength of CCA50 across the pre-processing alternatives is due to the grading change and the reduction in flakiness index of the fine aggregates, causing an increase in the packing density, Table 6. Compaction during casting has a positive influence on the concrete strength and is hindered by flaky aggregates in concrete [24]. Therefore, by reducing aggregate flakiness with pre-processing, more effective compaction takes place, which additionally contributes to the compressive strength of all concrete mixes in this study. Reference concrete strength is achieved by both CCA100 and CCA50 at MP15.

#### 3.3.3. Packing Density

**Figure 7:**
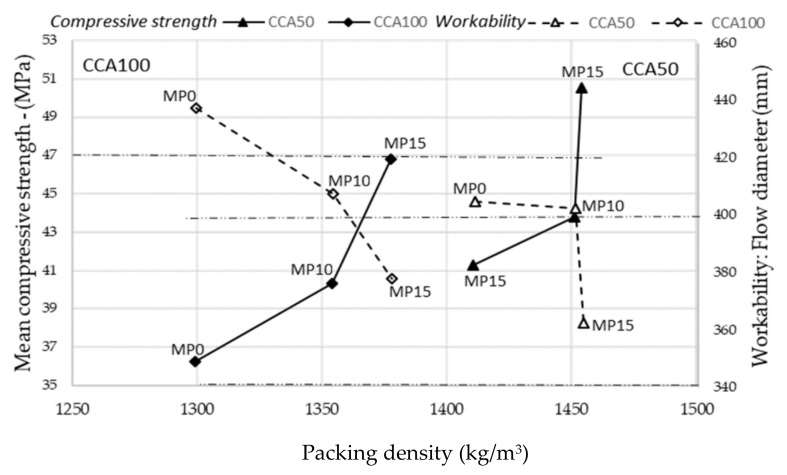
Packing density of aggregate related to compressive strength and workability; Reference compressive strength: 47 MPa; Reference workability: Class F2 (340–400 mm).

The packing density summarizes improvements in different aggregate properties arising from mechanical pre-processing and maybe seen as a property assessing CCA quality. Packing density is a good way to assemble the influences of different aggregate properties on the compressive strength and workability of the concrete. It may be interpreted as a bridging property between aggregate and concrete properties. This is ascertained for CCA50 and CCA100, which show the same tendencies for compressive strength and workability at different packing densities, see Figure 7. The compressive strength values are an average of three specimens, the workability stands for a single sample.

The stability of the relationship between packing density, workability, and compressive strength can be investigated for different concrete recipes. This relationship can be useful to pre-determine concrete properties arising from CCA of given quality shown by packing density.

## 4. Conclusions and Discussions

Two mechanical pre-processing durations are investigated on concrete mixes with two aggregate replacement percentages. Between the two pre-processing durations, 15 min pre-processing is noticed to be optimum since:

- Reduces the most adhered mortar content in all CCA fractions and improves the grading of fine CCA by bringing it closer to the reference aggregate.
- Significant improvements in flakiness index and shape index of coarse CCA.
- The pre-soaking technique is compatible with mechanical pre-processing in optimizing pre-soaking water amount resulting from the reduction in adhered mortar content by abrasion.
- Both compressive strength and workability of CCA50 and CCA100 are reaching compressive strength of reference concrete.
- The aggregate properties collectively influence the packing density, which increases with increasing pre-processing duration for both CCA replacements.
- Packing density is the property concluding the collaborative effect of several properties such as density, particle grading, flakiness index, shape index, unit weight, and void content.
- The packing density has similar influences for compressive strength and workability for both CCA concrete, the packing density maybe used to indicate CCA quality along with water absorption and other properties confirmed by further investigations.
- For further research, the stability of the observed relationship between packing density, workability, and compressive strength may be investigated for different concrete recipes.

## Figures and Tables

**Table 1:** Sieves for washing different aggregate fractions during mechanical pre-processing

**Sieve Mesh Size (mm)**	**Reference Concrete Fractions (mm)**		**CCA Concrete Fractions (mm)**		
	8/11.2	0/8	8/11.2	0.5/4	0/4
	4	0.063	4	0.8	0.063

**Table 2:** Aggregate properties investigated

Properties	Test Standards	Aggregate Fractions Tested	Samples
Test sampling	SS EN 932-1 [32]	Coarse, fine	-
Flakiness index	SS-EN 933-3:2012 SBUF 122270 [33]	Coarse Fine	2
Shape index	SS-EN 933-4:2008	Coarse	5
Particle grading	SS-EN 933-1:2012	Coarse, fine	2
Fineness modulus	SS-EN 12620 + A1:2008	Coarse, fine	-
Unit weight	ASTM C29/29M—17a, Method C—shovelling procedure	Coarse, fine	3
Void content			3
Apparent density	SS-EN 1097-6:2013	Coarse, fine	1
Water absorption	Modified pycnometer method [17]	Combined fraction	3
Packing density	Derived from unit-weight	Combined fraction	1

**Table 3:** Concrete recipes for mixes with and without mechanically pre-processed aggregates

		Reference Concrete			CCA50 Concrete			CCA100 Concrete		
		MP0	MP10	MP15	MP0	MP10	MP15	MP0	MP10	MP15
Cement	(kg/m^3^)	490								
Super plasticizer		3.7								
Pre-soaking water in CCA					24.08	24.09		44.86		36.2
Mixing water		245								
8/11.2 NA		729			729			-		
0/8 NA		845			-			-		
8/11.2 CCA		-			-			708.3		
0.5/4 CCA		-			519.4	157.4		550.9	157.4	
0/4 CCA		-			314.8	676.8		314.8	708.3	

**Table 4:** Packing density and grading of concrete mixes.

	Aggregate Fractions	Reference Concrete		CCA50			CCA100		
Pre-Processing Alternatives		0/8	8/11.2	0/4 CCA	0.5/4 CCA	8/11.2	0/4 CCA	0.5/4 CCA	8/11.2 CCA
MP0	Grading	53%	47%	20%	33%	47%	20%	35%	45%
	Packing density (kg/m^3^)	1672.7		1410.6			1299.4		
	Fineness modulus	4.48		4.83			4.56		
MP10	Primary grading	53%	47%	20%	33%	47%	20%	35%	45%
	Packing density (kg/m^3^)	1640.7		1405.4			1306.9		
	Fineness modulus	4.6		4.87			4.80		
	Modified grading			43%	10%	47%	43%	10%	47%
	Packing density (kg/m^3^)			1451.1 ↗			1354.1 ↗		
	Fineness modulus			4.69			4.59		
MP15	Primary grading	53%	47%	20%	33%	47%	20%	35%	45%
	Packing density (kg/m^3^)	1630.8		1421.6			1344.3		
	Fineness modulus	4.55		4.84			4.78		
	Modified grading			43%	10%	47%	43%	10%	47%
	Packing density (kg/m^3^)			1454.1 ↗			1377.6 ↗		
	Fineness modulus			4.57			4.48		

**Table 5:** Water absorption of combined CCA fractions before and after mechanical pre-processing

Combined Fractions	Water Absorption before Pre-Processing	Grading after Pre-Processing	Water Absorption after Pre-Processing	
	MP0		MP10	MP15
CCA 50 mean $\bar{x}$, standard deviation s for three samples	$\bar{x}$ = 2.8% s = 0.2%	Primary grading	$\bar{x}$ = 2.9% s = 0.5%	$\bar{x}$ = 2.6% s = 0.3%
		Modified grading	$\bar{x}$ = 3.6% s = 0.43	$\bar{x}$ = 3.6% s = 0.4%
CCA 100 mean $\bar{x}$, standard deviation s for three samples	$\bar{x}$ = 5.7% s = 0.4%	Primary grading	$\bar{x}$ = 4.6% s = 0.6%	$\bar{x}$ = 3.8% s = 0.3%
		Modified grading	$\bar{x}$ = 5.7% s = 0.4%	$\bar{x}$ = 4.5% s = 0.2%

**Table 6:** Test results of aggregate properties, packing density, and concrete density

Properties	Pre-Processing Alternatives	Reference Concrete Aggregate		CCA		
		0/8	8/11.2	0/4 CCA	0.5/4 CCA	8/11.2 CCA
Flakiness index (%) range of results for two samples	MP0	4.7 ± 0.5	18.3 ± 1.8	5.06 ± 0.04	3.4 ± 0.2	10.4 ± 0.6
	MP10	4.2 ± 0.4	15.4 ± 1	4.1 ± 0.8	3.4 ± 0.2	8.6 ± 0.5
	MP15	3.8 ± 0.7	15.8 ± 0.5	4.7 ± 0.3	3.5 ± 0.8	6.2 ± 0.5
Shape index (%) range of results for two samples	MP0	-	13.2 ± 1.8	-	-	12.5 ± 1.4
	MP10	-	10.6 ± 1.2	-	-	8.4 ± 1.1
	MP15	-	10.1 ± 0.7	-	-	6.2 ± 0.6
Unit weight (kg/m^3^) mean $\bar{x}$, standard deviation s for three samples	MP0	$\bar{x}$ = 1837 s = 7.6	$\bar{x}$ = 1486 s = 19.3	$\bar{x}$ = 1475 s = 6.6	$\bar{x}$ = 1263 s = 4.39	$\bar{x}$ = 1249 s = 4.84
	MP10	$\bar{x}$ = 1754 s = 12.2	$\bar{x}$ = 1512 s = 2.0	$\bar{x}$ = 1434 s = 35.1	$\bar{x}$ = 1312 s = 8.0	$\bar{x}$ = 1306 s = 9.7
	MP15	$\bar{x}$ = 1747 s = 2.69	$\bar{x}$ = 1498 s = 29.3	$\bar{x}$ = 1464 s = 7.6	$\bar{x}$ = 1299 s = 7.8	$\bar{x}$ = 1336 s = 4.8
Void content (%)	MP0	32	44.4	45.6	53.6	52.7
	MP10	34.3	43.3	45.9	49.8	50.1
	MP15	34.7	44.3	44.8	48.5	49.0
Apparent density (kg/m^3^)	MP0	2701	2720	2712	2720	2640
	MP10	2671	2665	2652	2615	2615
	MP15	2676	2694	2614	2527	2621
Packing density (kg/m^3^)	MP0	1672		CCA50/CCA100	1410/1299	
	MP10	1640		CCA50/CCA100	1451/1354	
	MP15	1630		CCA50/CCA100	1454/1377	
Hardened concrete density (kg/m^3^)	MP0	2402		CCA50/CCA100	2297/2116	
	MP10	2402		CCA50/CCA100	2298/2226	
	MP15	2413		CCA50/CCA100	2300/2232	
